# Biological roles of A-to-I editing: implications in innate immunity, cell death, and cancer immunotherapy

**DOI:** 10.1186/s13046-023-02727-9

**Published:** 2023-06-17

**Authors:** Jing Yuan, Li Xu, Hai-Juan Bao, Jie-lin Wang, Yang Zhao, Shuo Chen

**Affiliations:** 1 Department of Obstetrics and Gynecology, Department of Gynecologic Oncology Research Office, Guangzhou Key Laboratory of Targeted Therapy for Gynecologic Oncology, Guangdong Provincial Key Laboratory of Major Obstetric Diseases, The Third Affiliated Hospital of Guangzhou Medical University, No.63 Duobao Road, Liwan District, Guangzhou City, Guangdong Province 510150 P. R. China; 2 Department of Laboratory Medicine, The First Hospital of China Medical University, Shenyang, 110001 China

**Keywords:** A-to-I editing, Innate immunity, Cell death, Targeted therapy, Cancer immunotherapy

## Abstract

Adenosine-to-inosine (A-to-I) editing, a key RNA modification widely found in eukaryotes, is catalyzed by adenosine deaminases acting on RNA (ADARs). Such RNA editing destabilizes endogenous dsRNAs, which are subsequently recognized by the sensors of innate immune and other proteins as autologous dsRNAs. This prevents the activation of innate immunity and type I interferon-mediated responses, thereby reducing the downstream cell death induced by the activation of the innate immune sensing system. ADARs-mediated editing can also occur in mRNAs and non-coding RNAs (ncRNAs) in different species. In mRNAs, A-to-I editing may lead to missense mutations and the selective splicing of coding regions. Meanwhile, in ncRNAs, A-to-I editing may affect targeting and disrupt ncRNAs maturation, leading to anomalous cell proliferation, invasion, and responses to immunotherapy. This review highlights the biological functions of A-to-I editing, its role in regulating innate immunity and cell death, and its potential molecular significance in tumorigenesis and cancer targeted therapy and immunotherapy.

## Introduction

DNA and RNA modification are crucial for a number of biological processes, and post-transcriptional modifications to RNA, in particular, are being increasingly understood [1]. In recent years, with the continued development of sequencing technologies, over 170 different RNA modifications have been found, including pseudouridine (Ψ), 5-methylcytosine (m5C), N6-methyladenosine (m6A), 7-methylguanosine (m7G), and RNA editing with cytosine to uridine (C-to-U) and adenosine to inosine (A-to-I) [2].

**Fig. 1:**
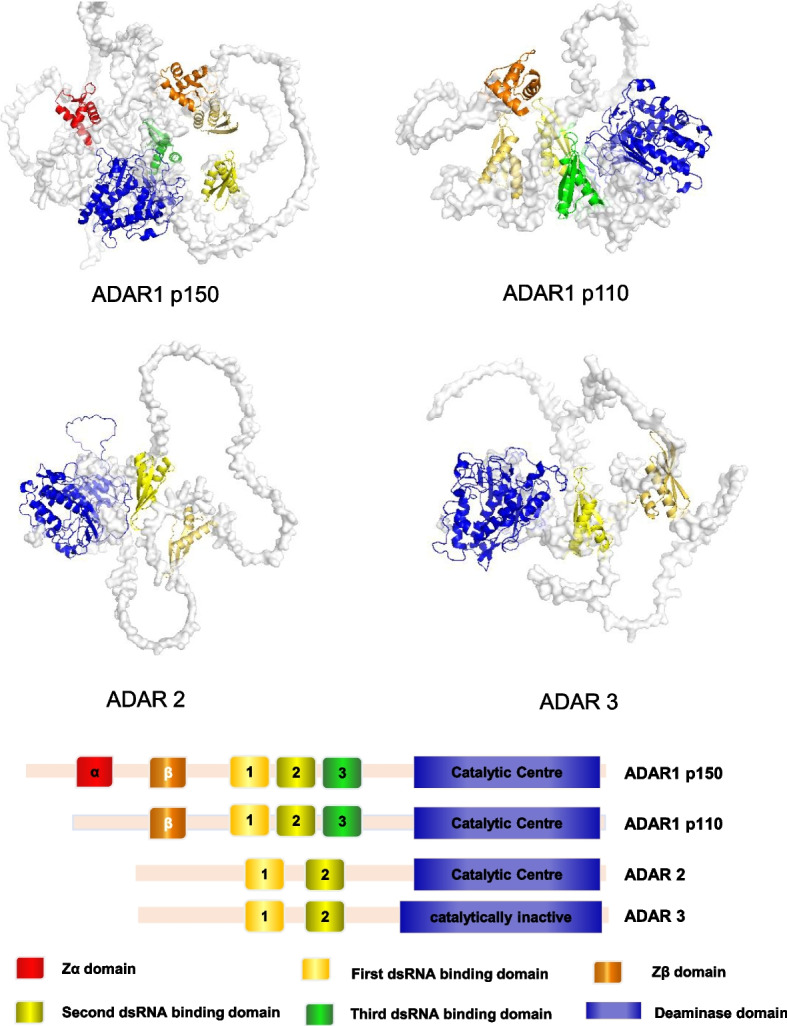
Schematic diagram of the structural domains and structures of ADAR family proteins

In mammals, A-to-I editing is one of the greatest commonly detected co-transcriptional/post-transcriptional RNA modifications. It is catalyzed by adenosine deaminases acting on RNA (ADARs), and unlike RNA methylation modifications (m6A, m5C), it is irreversible [3]. Moreover, the RNA editing can be detected directly by sequencing and does not require pull-down treatment with labeled antibodies or chemical treatment prior to sequencing. In the process of preparing the library for RNA-seq, the reverse transcription produces the first strand of complementary DNA (cDNA), which results in I transformation into G. Therefore, it is highly likely that A > G is a site of RNA editing. To date, several bioinformatics tools have been used to discover RNA editing sites from RNA-seq data, for example, GIREMI [4], JACUSA [5], REDItools [6–9], RES-Scanner [10], RNAEditor [11] and SPRINT [12]. ADARs were first detected in 1987 in the oocytes and eggs of the African clawed frog [13]. They were subsequently labelled as A-to-I editing enzymes that catalyze the C6 hydrolytic deamination of adenosine to inosine in double-stranded RNA (dsRNA), creating functional A-to-G mutations [14]. Three ADAR proteins have been discovered in humans: ADAR1, ADAR2, and ADAR3. Each of these proteins has a deaminase domain and multiple dsRNA binding domains (dsRBDs). While ADAR2 and ADAR3 have two dsRBDs, ADAR1 has three [15]. At the C-terminus of all proteins there is a deaminase domain, which serves as the catalytic centre of the protein. Meanwhile, the dsRBDs mediate dsRNA binding and homodimerization. In ADAR1, the third dsRBD includes a nuclear localization sequence (NLS), whereas in ADAR2, the same sequence is found at the N-terminal. There are two subtypes of ADAR1: ADAR1 p110, which is short and constitutively expressed, and ADAR1 p150, which is long and inducible by interferon (IFN) [16]. ADAR1 p110 includes a Z-DNA binding domain Zβ at the N terminus, whereas ADAR1 p150 contains a Zβ and a Zα domain with a nuclear export sequence (NES) [17] (Fig. 1). Both ADAR1 p110 and ADAR2 are almost entirely found in the nucleus of the cell. ADAR1 p150 has an NLS and NES and can shuttle between the nuclear and cytoplasm, but it functions mainly in the cytoplasm [18]. ADAR1 is widely expressed in almost all tissues. However, ADAR2 is more specifically expressed in regions such as the brain, lungs, and arteries and induces a high rate of RNA editing in neuronal cells. Meanwhile, ADAR3 is specific to the brain. Although it can inhibit other ADARs, it has no detectable editing function [19].

ADAR1 knockout (KO) (ADAR1-/-) mice have a phenotype that is embryonically deadly with significantly elevated protein levels of type I interferon IFN-α and IFN-β in embryonic tissue and undetectable type II interferon IFN-γ [20]. This indicates that ADAR1 effectively prevents endogenous dsRNAs identification by dsRNA sensors such as melanoma differentiation-associated gene 5 (MDA5), suppressing innate immunity and type I interferon-mediated responses [21]. Recent research has also demonstrated that ADAR1 controls immunity by limiting Z-type dsRNA (Z-RNA) buildup. The buildup of Z-RNA caused by the depletion or mutation of ADAR1 activates the Z-RNA sensor, Z-DNA binding protein 1 (ZBP1), which in turn triggers receptor interacting serinethreonine protein kinase 3 (RIPK3)-mediated necroptosis and PANoptosis [22] (Fig. 3). ADAR1 guards the body against a number of type I interferon activation-induced conditions such as autoimmune Aicardi-Goutières syndrome [23], and psoriasis [15]. Unsurprisingly, besides preventing autoimmune illnesses, ADAR1 also plays a role in cancer immunity [24].

**Fig. 2:**
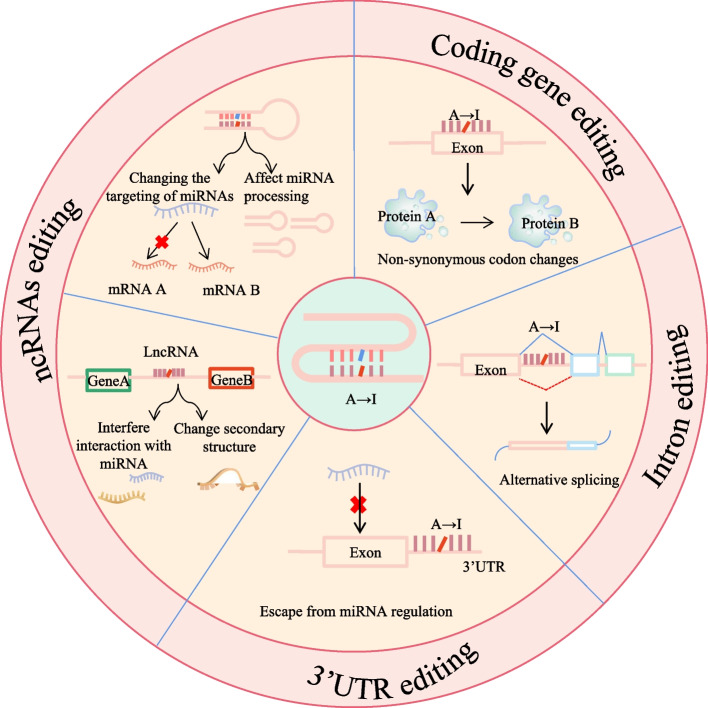
Schematic representation of the functional consequences that result from A-to-I editing acting on mRNAs versus ncRNAs

ADARs-mediated editing is critical to mammalian survival, and its misregulation may a contributor to tumorigenesis and progression. The RNA editing occurs at different locations and produces different effects. As inosine is mistaken for guanosine during base pairing, A-to-I editing in exonic regions can be the result non-synonymous mutations [25]. In addition, such editing can also occur in introns or 3' untranslated regions (3' UTRs), which control the expression of related coding regions [26]. Furthermore, the editing of non-coding RNAs (ncRNAs; mainly microRNAs [miRNAs] and long ncRNAs [lncRNAs]) affects their maturation and targeting [27] (Fig. 2). Overall, tumor cells support the progression of cancer by capitalising on the diversity introduced by A-to-I editing in terms of both transcriptomics and proteomics.

Studies have shown that ADARs-mediated editing has important functional and biological significance in processes such as innate immunity, cell death and tumorigenesis. Such editing affects the expression and activity of many transcripts, and abnormal editing is also closely associated with the development of many cancers. Several studies have shown that the RNA editing mainly inhibits the dsRNA-sensing pathway, leading to cancer cell immune tolerance [28–31], which leads to the development of cancer and lays the foundation for immunotherapy of tumors. In this review, we focus on the biological functions of A-to-I editing, the role in regulating innate immunity and cell death, and the potential molecular significance in tumorigenesis and cancer targeted therapy and immunotherapy. We also highlight the issues that remain to be addressed in this field and provide prospects for further research.

## Nature of A-to-I editing

A-to-I editing is a key RNA modification found widely in eukaryotes and is catalyzed by ADARs. The inosine formed via deamination exhibits altered hydrogen bonding patterns and readily pairs with cytosine bases, which are decoded as guanine [14]. An essential target for A-to-I RNA editing is dsRNAs derived from inverted Alu repeat elements (Alu dsRNAs). Owing to the abundance of these elements in human genomes, there are greater than 100 million A-to-I editing sites in the human transcriptome [32]. Wobble pairing, as opposed to Watson–Crick pairing, is observed between the resulting I-U base pairs when A-to-I editing happens at A-U base pairs in dsRNAs, which strains the double helix structure and destabilizes the dsRNAs [33]. The inosine-containing endogenous dsRNAs are subsequently recognized by the sensors of innate immune and other proteins as an autologous dsRNAs, preventing the activation of innate immunity. Further, A-to-I editing affects transcript stability, mRNA localization, and the interaction of RNA with cellular pathways. A-to-I editing affects RNA stability in a direct or indirect manner. In lung adenocarcinoma, focal adhesion kinase (FAK) protein abundance is increased, mainly because ADAR1 binds to FAK transcripts and edits their specific intron sites to improve FAK mRNA stability [34]. It is thus clear that ADARs-mediated can directly affect RNA stability. The RNA editing can also affect RNA stability by recruiting the human antigen R (HuR, gene name ELAVL1) protein. In coronary atherosclerosis, cathepsin S (CTSS) transcripts contain inverted Alu repeat sequence regions, and edited Alu dsRNA disrupts the structure of dsRNA, and ADAR1 recruits the RNA-binding protein HuR, thereby increasing the stability of CTSS mRNA [35]. Notably, it can also enhance transcriptional splicing and directly alter the amino acid sequence in open reading frames. Dysfunctional A-to-I editing is associated with multiple disease conditions, including autoimmune disease and cancer.

## A-to-I editing in innate immunity

Innate immunity is host's first defence against foreign agents such as viruses, and its dynamic balance is key for maintaining homeostasis. When the body is invaded by foreign pathogens or abnormal endogenous nucleic acids (NAs) are present, pattern recognition receptors (PRRs) are enriched on innate immune cells such as dendritic cells (DCs). PRRs sense various damage-associated molecular patterns (DAMPs) and pathogen-associated molecular patterns (PAMPs) and initiate signaling cascades that up-regulate a variety of immune genes, including those that encode for inflammatory cytokines and chemokines. In particular, type I IFN is upregulated [36]. An inflammatory antiviral cellular state is established when IFN is generated, which in turn stimulates the transcription of IFN-stimulated genes (ISG) [37, 38]. Even though IFN signaling is necessary for the prevention and treatment of infections, aberrant IFN signaling can lead to the development of pathological inflammation. Unlike other PAMPs, abnormal endogenous NAs, such as short interspersed nuclear elements (SINEs) like Alu dsRNAs, are produced endogenously and are highly abundant in the host. They can activate dsRNAs sensors and trigger an innate immune reaction, leading to the aberrant production of IFN and subsequent pathological effects. Hence, the body prevents the activation of innate immunity by dsRNAs through A-to-I editing, the degradation of Alu RNA by endonucleases, and the chelation of Alu RNA by RNA-binding proteins [39]. Here, we focus on how A-to-I editing hinders PRRs from sensing endogenous dsRNA and thereby activate innate immunity.

In humans, ADAR1 p150, ADAR1 p110, and ADAR2 have A-to-I editing activities. Although all three enzymes can prevent the dsRNA-induced activation of innate immunity via A-to-I editing, they regulate innate immunity differently. This is probably due to the differences in their domain and localization. ADAR1-/- mice, ADAR1 p150 specific KO (ADAR1 p150-/-) mice, and ADAR1 carrying the editing point mutation E861A (ADAR1 E861A/E861A) mice all show lethality to embryos. These mutations result in large-scale apoptosis and the increased expression of ISG [20, 21, 40–42]. Notably, in these ADAR1 mutant mice, simultaneous knock down IFIH1-encoded MDA5, or knock down of its downstream mitochondrial antiviral signalling protein (MAVS) prevents embryonic death and ameliorates ISG overexpression [43, 44]. MDA5 belongs to the RIG-I-like receptor family and is a cell membrane sensor for viral dsRNA. By activating MAVS, it sets of antiviral reactions like the induction of ISGs [45]. Recent studies have demonstrated that ADAR1 p110 specific KO (ADAR1 p110-/-) and ADAR2 specific KO (ADAR2-/-) mice show no upregulation of ISGs. This indicates that ADAR p150-induced RNA editing is key for preventing MDA5 from recognizing endogenous dsRNAs as nonself.

ADAR1 p150 also has a unique Zα domain that promotes A-to-I editing of endogenous Alu elements and prevents pairing of inverted Alu repeat sequences to form dsRNAs. Thereby, it reduces the accumulation of endogenous Z-NAs, inhibiting the PANoptosis and necroptosis induced by the Z-RNA sensor ZBP1 [46]. PANoptosis is an inflammatory programmed cell death regulated by the PANoptosome complex with key features of pyroptosis, apoptosis or necroptosis. PANoptosis cannot be characterized by any of the pyroptosis, apoptosis and necroptosis modes of death alone [47]. Both the Zα domain of ADAR1 p150 and its editing activity are required to control the total amount of endogenous Z-NAs. In Adar1-/- mouse embryonic fibroblast (MEF) cells, ADAR1 p150 (N175A/Y179A) expressing a mutation in the Zα structural domain completely inhibited Z-RNA accumulation upon IFN induction, whereas ADAR1 p150 E861A/E861A partially inhibited Z-RNA accumulation [22].

**Fig. 3:**
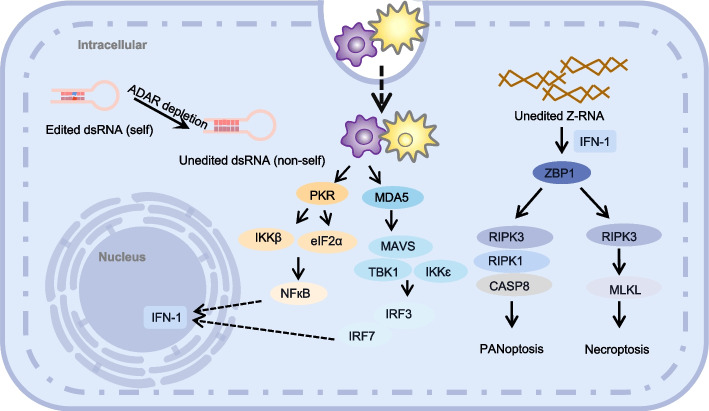
The role of ADAR1 in regulating innate immune and cell death responses to dsRNA and Z-RNA: Upon ADAR1 depletion, unedited dsRNA triggers the pattern recognition receptor MDA5, PKR and unedited Z-RNA triggers ZBP1, both ultimately leading to interferon-induced and apoptotic antiviral mechanisms

Protein kinase R (PKR), coded by EIF2AK2, is a type I IFN-induced antiviral protein that also recognizes dsRNAs and is involved in innate immunity. Unlike MDA5, PKR does not cause ISG upregulation, which primarily leads to the inhibition of translation initiation and cell growth [48, 49]. Stress particles can be co-located with ADAR1 and PKR in cells infected with measles virus. Stress granules that is the accumulation of stagnant translation complexes and RNA binding proteins in cytoplasm are PKR-dependent and inhibited by ADAR1 [50]. Furthermore, not only does ADAR1 p150 contribute to the suppression of PKR activation, but so does its p110 isoform [51] (Fig. 3).

Although there is no direct evidence that the ADAR2-mediated editing is directly involved in innate immune responses, recent studies have suggested that ADAR2 may like ADAR1, prevent RNA sensors such as MDA5 and PKR from recognition of endogenous dsRNAs as nonself entities. ADAR2-mediated editing allows the viral RNA of the Borna disease virus (BoDV), an RNA virus that replicates in the nucleus, to appear as "self" RNA. Hence, this virus can escape innate immune reaction of the host and establish a long-lasting infection in the nucleus. In human embryonic oligodendrocytes, ADAR2 KO (ADAR2-/-) enhances the BoDV-induced immune response and increases the expression of the pro-inflammatory molecules Interleukin-6 (IL-6) and recombinant human C-X-C motif chemokine 10 (CXCL10) in the body. These effects are ameliorated by the overexpression of wild-type ADAR2 [52].

## A-to-I editing in cell death

ADAR1-/- and ADAR1 E861A/E861A mice die in utero 12.5 and 13.5, respectively [21, 40, 42]. The lethality can be attributed to failed fetal liver hematopoiesis, and mainly impaired erythropoiesis, and can be recapitulated in mice with restricted knock down of ADAR1 in the erythroid lineage [53]. Death of ADAR1-deficient mouse embryos or ADAR1-deficient cells can be prevented by deleting MDA5 or MAVS [40, 43, 44], suggesting that cell death occurs downstream of the trigger of the innate immune sensing system. It was shown that MDA5 activates IFN regulatory factors IRF3 and IRF7 in response to dsRNA by initiating the assembly of MAVS filaments, thereby increasing ISG expression [54]. The MAVS microfilaments also induce nuclear factor Kappa B subunit (NF-kb)-dependent tumor necrosis factor (TNF) expression by binding to the TNF receptor-associated factors TRAF3 and TRAF6. TRAF6 inhibits ISG responses by ubiquitinating IRF3, which in turn activates B cell lymphoma 2 (BCL2)-associated X (BAX), a regulator of apoptosis. When BAX forms a dimer with BCL2 homologous antagonist/killer (BAK) or ubiquitinated IRF3 (but not a protective BCL2 family member), apoptosis is triggered via mitochondrial pathways, resulting in cell death [54]. However, it has also been shown that the depletion of BAX and BAK does not prolong the survival of ADAR1 editing-deficient embryos, suggesting that MDA5-triggered cell death is not dependent on the intrinsic apoptotic pathway [55]. Hence, whether the process of cell death triggered by MDA5 after ADAR1 deletion or ADAR1 editing defects requires an intrinsic apoptotic pathway remains controversial and warrants further exploration.

Although simultaneous deletion of MDA5 or MAVS can reverse the embryonic lethality seen in ADAR1 p150-/- mice, ADAR1 p150/Ifih1 and ADAR1 p150/Mavs double-KO mice have high postnatal mortality rates [40, 44, 56, 57]. This suggests that ADAR1 p150 can also induce cell death through pathways other than the MDA5 pathway. Interestingly, the survival of ADAR1 p150/Ifih1 double-KO mice is prolonged if ZBP1 is concurrently deleted [56], suggesting that the cell death caused by ADAR1-/- may also be due to ZBP1-induced PANoptosis and necroptosis. In the absence of ADAR1 p150, Z-RNA can activate the ZBP1 protein, induce RIPK3-MLKL(mixed lineage kinase domain-like protein) to form necrosomes, cause MLKL oligomerization and phosphorylation, and promote the translocation of phosphorylated MLKL to the cell membrane. This causes membrane damage and eventually results in cell death [58] (Fig. 3).

In addition to ADAR1-mediated editing abnormalities, ADAR2-mediated editing abnormalities also cause cell death. In mice, the deletion of ADAR2 or defective ADAR2-mediated editing lead to postnatal death due to epilepsy [59]. After ADAR2 deletion, mouse motor neurons express unedited GluA2 at the Q/R site. The α-amino-3-hydroxy-5-methyl-4-isoxazole-propionicacid (AMPA) receptors containing unedited GluA2 show abnormal Ca^2+^ permeability, leading to progressive neuronal death [60]. In addition to acting on mRNAs to regulate cell death, ADAR2 can also act on ncRNAs. In one study, after miRNA-379-5p was subjected to ADAR2-mediated editing, its target was found to have changed from PTK2 to CD97. The edited miRNA-379-5p knocked down CD97, promoted caspase-mediated apoptosis, and inhibited cell proliferation [61].

## A-to-I editing in cancer

A-to-I editing levels are frequently elevated in several types of cancer, including hepatocellular carcinoma, non-small cell lung cancer, thyroid cancer, pancreatic cancer, esophageal cancer, cervical cancer, and multiple myeloma [3, 62–66]. In contrast, reduced levels of A-to-I editing are observed in metastatic melanoma, invasive breast cancer, and renal cancer [67, 68]. As described previously, ADAR-mediated editing at different locations produces different effects. A-to-I editing in the coding region can result in non-synonymous mutations, while the editing in introns or the 3' UTR can alter the expression levels of related coding regions. In ncRNAs, such as editing affects ncRNAs maturation and targeting (Fig. 2). In most cases, increased levels of the editing promote cancer development and progression; however, in some cancers, reduced levels of the editing mediate the cancer phenotype [67, 69]. This section focuses on how A-to-I editing affects tumorigenesis through four pathways: coding genes, introns, 3′ UTRs, and ncRNAs (Table 1).

**Table 1:** A-to-I editing in different tumours

Cancer	Gene	Protein	Substrate type	Editing residue (s)	References
Hepatocellular carcinoma	AZIN1	Antizyme inhibitor 1	Coding gene	S/G	[62]
	BLCAP	Bladder cancer-associated protein	Coding gene	Y/C	[68]
	ITGA2	Integrin alpha 2	Coding gene		[70]
	circARSP91		circRNA	circRNA	[71, 72]
Esophageal squamous cell carcinoma	AZIN1	Antizyme inhibitor 1	Coding gene	S/G	[73]
Colorectal carcinoma	AZIN1	Antizyme inhibitor 1	Coding gene	S/G	[74, 75]
Gastric cancer	hsa_circ_0004872		circRNA	circRNA	[76]
Pancreatic ductal adenocarcinoma	circNEIL3		circRNA	circRNA	[77]
Non-small-cell lung cancer	AZIN1	Antizyme inhibitor 1	Coding gene	S/G	[65]
Lung adenocarcinoma	FAK	Focal adhesion kinase	Intron		[34]
Multiple myeloma	GLI1	Glioma-associated oncogene 1	Coding gene	R/G	[78]
	NEIL1	Endonuclease 8-like 1	Coding gene	K/R	[79]
Melanoma	CCNI	Cyclin I	Coding gene	R/G	[24]
	miR-455-5p		miRNA	miRNA	[69]
	miR-378a-3p		miRNA	miRNA	[80]
	miR-222		miRNA	miRNA	[81]
	miRNA-149-3p		miRNA	miRNA	[82]
Breast invasive carcinoma	GABRA3	Gamma-aminobutyric acid receptor subunit alpha-3	Coding gene	I/M	[67]
	ARHGAP26	RhoA GTPase activating protein 26	3′ UTR	3′ UTR	[83]
	DHFR	Dihydrofolate reductase	3′ UTR	3′ UTR	[84]
	DFFA	DNA fragmentation factor alpha	3′ UTR	3′ UTR	[27]
	LINC00944		LncRNA	lncRNA	[85]
Cervical squamous cell carcinoma and endocervical adenocarcinoma	BLCAP	Bladder cancer-associated protein	Coding gene	Y/C; Q/R; K/R	[86]
Thyroid cancer	CDK13		Coding gene	Q/R	[87, 88]
	miR-200b		miRNA	miRNA	[89]
Prostate adenocarcinoma	PCA3	Prostate cancer antigen 3	LncRNA	Duplex with PRUNE2	[90]
Chronic myelogenous leukemia	pri-let-7d				[91]

### Coding genes

Genes known to undergo A-to-I editing include antizyme inhibitor 1 (AZIN1), bladder cancer-associated protein (BLCAP), integrin alpha 2 (ITGA2), glioma-associated oncogene 1 (GLI1), endonuclease 8-like 1 (NEIL1), cell cycle protein I (CCNI), gamma-aminobutyric acid receptor subunit alpha-3 (GABRA3), and CDK13. Of these, AZIN1 [62, 65, 73–75], BLCAP [68, 86], ITGA2 [70], GLI1 [78], NEIL1 [79], and CDK13 [87, 88] after the editing promote cancer development. CCNI [24], GABRA3 [67] after the editing can inhibit tumor cell growth, invasion and migration. AZIN1 and BLCAP have been shown to promote tumorigenesis in various cancers.

Edited AZIN1 promotes tumor cell proliferation, invasion, and migration in a wide range of cancers, including hepatocellular carcinoma [62], non-small-cell lung cancer [65], colorectal cancer [74, 75], and esophageal squamous cell carcinoma [73]. For example, in hepatocellular carcinoma, the conversion of serine (S) to glycine (G) at residue 367 of the AZIN1 β-15 chain alters the protein's conformation, inducing cytoplasmic to nuclear translocation and subsequent tumor development [62]. Although BLCAP exhibits tumor-suppressive effects in various cancers [92], edited BLCAP mainly exerts pro-cancer effects. In hepatocellular carcinoma, the edited BLCAP gene enhances the phosphorylation of AKT, mTOR, and MDM2 and inhibits the phosphorylation of TP53, thereby promoting cell proliferation [68].

In contrast to the above examples, low levels of A-to-I editing are associated with poor prognoses in some cancers [24, 67]. The R75G peptide from ADAR1-edited CCNI stimulates tumor-infiltrating lymphocytes (TILs) in melanoma and promotes the TIL-induced destruction of cancer cells [24]. The expression of edited GABRA3 on the cell surface is reduced in non-invasive breast cancer, which prevents AKT activation and thereby prevents breast cancer cells from migrating, invading, and metastasizing [67].

### Introns

So far, for the editing of introns in the coding region in tumors, focal adhesion kinase (FAK) is more frequently studied. A-to-I editing of the FAK intron region leads to increased transcript stability. In lung adenocarcinoma, the editing of specific intron sites on chr8:141,702,274 in the FAK transcript increases the stability of FAK mRNA and expression of the FAK protein, thereby promoting tumor mesenchymal properties, migration and invasion [34].

### 3′ UTRs

Generally, the 3′ UTRs of RhoA GTPase activating protein 26 (ARHGAP26), DNA fragmentation factor alpha (DFFA), and dihydrofolate reductase (DHFR) are most commonly studied in the context of A-to-I editing. These edited 3′ UTRs are mainly found in breast cancer. The editing of the 3′ UTR affects the regulation of these genes through two primary methods. The most common is the A-to-I editing of Alu dsRNA in the 3′ UTR, which — when unedited — binds to and is regulated by miRNAs. The other approach relies on editing to alter the stability of mature mRNAs, primarily via the recruitment of HuR proteins to ADAR1. Several studies have established that RNA editing in the 3′ UTR can create or disrupt miRNA binding sites, thereby altering the stability of cancer-associated mRNAs. When DHFR mRNA is A-to-I edited in breast cancer, it does not bind to miR-25-3p and miR-125a-3p. Therefore, translation is not disturbed, and the elevated levels of DHFR mRNA and protein enhance cell proliferation and methotrexate resistance [84]. Altered RNA stability is an important mechanism for A-to-I editing-mediated regulation of gene expression. ADAR1 recruits and interacts with with HuR, a family of RNA-binding proteins, which selectively bind to single-stranded AU-rich RNA sequences to increase the stability of transcripts [16].

### ncRNAs

A-to-I editing primarily modifies the targeting and maturation of ncRNAs and thereby affects their role in cancers. In melanoma, A-to-I editing by ADAR1 reduces the ability of pri-miR-455 to bind to Drosha and get processed into mature miR-455-5p, which prevents miR-455-5p from promoting melanoma growth and metastasis in vivo [69]. When miRNA-379-5p is subjected to A-to-I editing by ADAR2, its target switches from PTK2 to CD97. The knockdown of CD97 can increase cysteine-mediated apoptosis, and the edited miRNA-379-5p binds to the CD97 (DNA) 3' UTR, thereby downregulating the mRNA and protein levels of CD97, promoting apoptosis, and inhibiting cell proliferation and tumor growth [61].

## A-to-I editing: a potential tool for tumor targeted therapy and immunotherapy

### Targeted therapy

In most tumors, elevated levels of A-to-I editing promote tumorigenesis and development. For example, AZIN1 and BLCAP promote cancer development after editing [62, 68, 73–75]. But in metastatic melanoma and invasive breast cancer, tumorigenesis and development are inhibited instead after elevated levels of A-to-I editing. For example, CCNI and GABRA3 are edited to inhibit tumor cell growth, invasion and migration [24, 67]. Recent studies have shown that site-directed RNA editing (SDRE) using ADAR can modulate the level of transcript editing and control the selectivity of editing through guanosine mismatches [93]. Unlike DNA editing, A-to-I editing does not permanently modify the genome, with significant safety advantages and relatively minor consequences of any off-target editing that occurs [94]. However, A-to-I editing still has the problem of off-target editing. Research has shown that GOTI (genome-wide off-target analysis by two-cell embryo injection) is capable of detecting off-target mutations in DNA editing by editing one oocyte of two-cell mouse embryos with CRISPR-Cas9 or base editors [95, 96], but whether it can be applied to A-to-I editing remains further investigation.

**Fig. 4:**
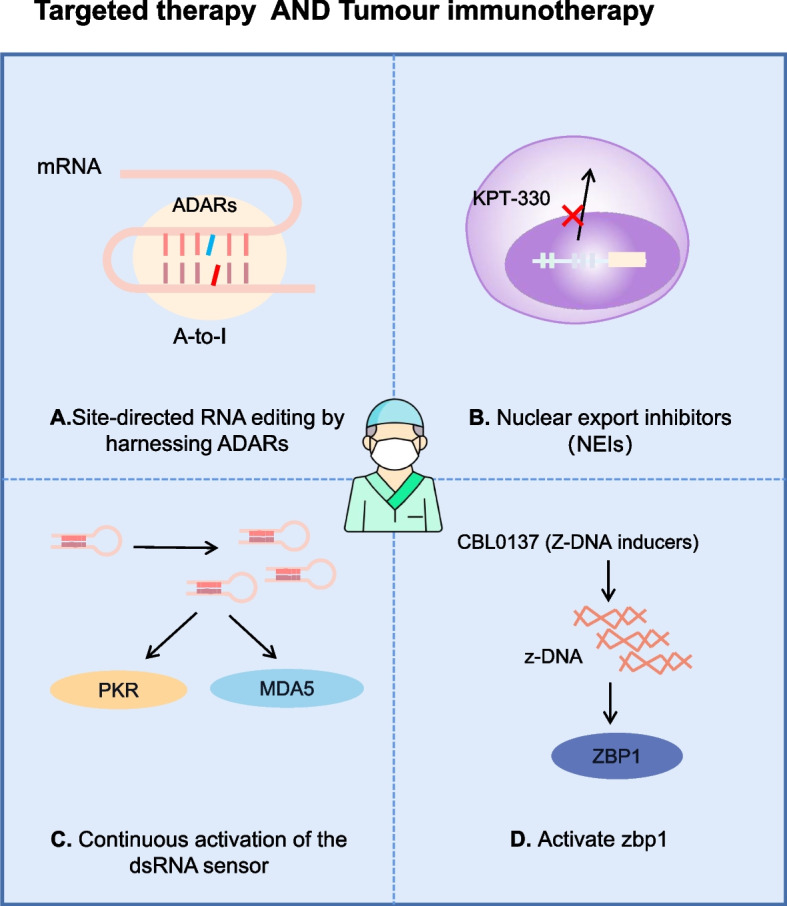
Potential targeted therapies for A-to-I RNA editing in cancer and tumour immunotherapy: **A** Site-directed RNA editing by harnessing ADARs; **B** Nuclear export inhibitors (NEIs) such as KPT-330 sequester ADAR1p150 in the nucleus; **C** Anti-cancer epigenetic inhibitors induce transcription of repetitive sequences that form dsRNAs and continuously activate the dsRNA sensor pathway; **D** Z-DNA inducer CBL0137 induces the formation of Z-DNA and activates ZBP1

Site-directed RNA editing requires a guide RNA (gRNA) that directs the ADAR to the target site for targeted editing. The most commonly used gRNA is an antisense oligonucleotide (ASO) that uses its single-stranded RNA structural domain to bind to the target mRNA through base pairing, while other structural domains take up the ADAR into the RNA and the ADAR converts adenosine A to inosine I. ADAR is further divided into endogenous and exogenous. Exogenous ADAR proteins or their catalytic structural domains are fused to λ-phage N protein (λN peptide) [97–100], SNAPtag [101–105] or CRISPR-Cas [106, 107], and chimeric ADAR proteins (such as SNAP-ADAR) are carried to the target editing site for editing using gRNA. Both LEAPER and CLUSTER can recruit endogenous ADAR for targeted editing, but their gRNAs differ. The gRNA of LEAPER is an ADAR-recruiting RNA (arRNA) [108, 109], whereas gRNA for CLUSTER is a cluster guide RNA (CLUSTER gRNA) [110]. Stafforst's team also tested the effect of anti-bases (U, C, A, G) on editing yield and found that U and C gave quantitative yields, but editing with adenosine as the anti-base was less efficient and editing with guanosine as the anti-base was severely hampered [102]. The overall picture is that the application of A-G mismatches reduces off-target editing events and A-C mismatches are applied to improve target editing efficiency. In tumors, applying A-G mismatches to inhibit editing or applying A-C mismatches to promote editing and restore transcript editing levels to normal would be a new therapeutic strategy with minimal systemic effects and maximum therapeutic benefit (Fig. 4A).

### Tumor immunotherapy

Tumors are classified as hot or cold based on the infiltration of immune cells and surrounding cells. As hot tumors show infiltrating immune cells, immunosuppressants are effective against them. Meanwhile, cold tumors are immune cell-suppressive tumors, and immunotherapy is less effective against them. However, immune checkpoint blockade (ICB) therapy offers new avenues for cold tumor immunotherapy. Nevertheless, tumors that do not respond to ICB and are resistant to immunosuppression still exist. Recent studies have demonstrated that ADAR1 loss-of-function makes cancer cells more sensitive to immunotherapy, reducing immune tolerance and sensitivity to ICB agents [111]. Notably, RNA editing enzymes can inhibit immunogenic dsRNAs and endogenous Z-RNA to suppress the ICB response.

The dsRBDs of ADAR1 bind to promiscuous dsRNAs and perform A-to-I editing of the dsRNAs. As a result, the dsRNAs cannot bind to dsRNAs sensors (MDA-5 and PKR). This inhibits IFN production and ISG expression [30, 111, 112], establishing a mechanism for evading recognition by the immune system and enhancing tumor malignancy. Recent studies have shown that ADAR1 deletion increases the susceptibility of tumors to ICB therapy. Lei's team developed a genetically engineered nanoparticle, siADAR1-LNP@mPD1, that can block the PD1/PDL1 immunosuppressive axis by presenting PD1 protein on the envelope. In addition, siADAR1 can be effectively delivered to cancer cells through nanoparticles designed to silence ADAR1 expression, leading to increased production of type I interferon, making cancer cells more sensitive to secreted effector cytokines and significantly halting cell growth [113]. In mouse melanoma B16 cells (a mouse model of human melanoma), a significant increase in CD8 + T cells and enhanced killing were observed in ADAR1-deficient (Adar1-null) tumors when compared with control tumors. The upregulation of genes associated with CD8 + T cell activation and effector functions was also detected [111]. Further studies have revealed elevated levels of antiviral cytokine and chemokine expression in Adar1-null tumor cells in response to IFN stimulation. MDA5 and PKR-mediated enhancement of sensitivity of Adar1-deficient tumors to antitumor immunity by different mechanisms. In the context of ADAR1 deletion, Ifih1 (MDA5) gene inactivation is followed by the suppression of IFN secretion, and MDA5 inactivation leads to only a slight increase in inflammation when compared with the inactivation of other genes, such as Ddx58 (RIG-I), Mavs (MAVS), and Eif2ak2 (PKR). This suggests that MDA5 activation increases IFN-I production and promotes inflammation and immune infiltration. The activation of PKR leads to translation inhibition and growth arrest, and in ADAR1-silenced cancer cells, cell viability is restored only after the concomitant deletion of PKR [30, 51, 112]. Overall, the sustained activation of the dsRNA sensor pathway following ADAR1 deletion reduces cancer cell viability, as demonstrated by the application of anti-cancer epigenetic inhibitors capable of inducing the transcription of repetitive sequences that form dsRNAs [29, 114] (Fig. 4C).

The Zα structural domain of ADAR1p150 recognizes and binds Z-RNA or Z-DNA and performs the A-to-I editing of the Z-RNA. The modified Z-RNA is not sensed by ZBP1, thereby inhibiting ZBP1-induced PANoptosis, which promotes tumorigenesis [22, 115]. The knockdown of ADAR1 in immortalized wild-type MEFs using CRISPR results in greater IFN-mediated Z-RNA accumulation and the increased intranuclear co-localization of ZBP1 with Z-RNA. It also increases the interaction of ZBP1 with RIPK3 and MLKL and phosphorylation activation level of MLKL, indicating that ADAR1 knockdown can inhibit tumor growth by promoting Z-RNA accumulation and activating ZBP1-mediated programmed cell necrosis [22]. This evidence suggests that ADAR1 may be a potential target for tumor therapy. Although no inhibitors of ADAR1 exist, nuclear translocation inhibitors such as KPT-330 and leptomycin B have been shown to sequester ADAR1p150 in the nucleus in combination with IFNs and to activate ZBP1 via Z-RNA induction [115] (Fig. 4B). Additionally, in the B16-F10 and YUMMER1.7 mouse models of malignant melanoma, combined treatment with CBL0137 (an activator of ZBP1) and an anti-PD-1 antibody (an immune checkpoint inhibitor) has been found to induce tumor regression [22] (Fig. 4D).

## Conclusion and outlook

A‑to‑I editing is one of the greast important epigenetic modifications in mammals. Unlike other epigenetic modifications, ADAR-mediated editing is irreversible. It has important roles in innate immunity, cell death, and tumor progression. Its dysregulation can result in neurological and developmental defects, autoimmune diseases, and malignancies, and the mechanisms involved in these pathological processes are multifaceted. A-to-I editing primarily affects the stability of dsRNAs, preventing them from being recognized by dsRNAs sensors. This inhibits IFN production, suppressing innate immunity and also reducing cell death downstream of innate immune system activation. In human malignancies, the editing affects the expression and activity of many transcripts, which in turn affect important oncogenes and related regulators. Whether A-to-I editing is a driver of cancer progression remains an open question. Nevertheless, it is clear that A-to-I editing is an important marker of tumor development and a key target for tumor therapy. The role of A-to-I editing in tumors and other diseases is an exciting area of research. The instantaneous targeted modification of a specific RNA is extremely appealing. Such targeted modification technology holds the promise of becoming a practical therapeutic tool for cancers and other diseases.

RNA editing technology is a transient alteration of mRNA, which does not change the genome sequence, and involves fewer safety and ethical issues than DNA editing technology, but RNA editing technology can still produce off-target effects, generating unexpected products or effects that may pose a risk of modifying the biological environment to the point of threatening the health of future generations of human beings and the survival of human beings. Before targeted RNA editing technology can be used in clinical practice, in addition to solving the main off-target problem, the following problems need to be solved: 1) the exogenous protein may cause the body to produce an immune response; 2) the fusion protein may have a neutralization reaction with the body's antibodies resulting in editing failure. The development of new gRNAs and delivery strategies for specific tissues or organs will be the main research and development areas for RNA editing technology routes. Improving the efficiency of A-to-I editing and reducing the minimum dose to achieve an effective therapeutic effect is likely to reduce the occurrence of side effects such as immune reactions. Although RNA editing technology is still in the preclinical stage, it has shown promising applications in several fields such as oncology and is expected to become a hot spot for the next generation of precision medicine.

## Data Availability

The data that support the findings of this study are available from the corresponding author upon reasonable request.

## Abbreviations

A-to-I: Adenosine-to-inosine
ADARs: Adenosine deaminases acting on RNA
ncRNAs: Non-coding RNAs
Ψ: Pseudouridine
m5C: 5-Methylcytosine
m6A: N6-methyladenosine
m7G: 7-Methylguanosine
C-to-U: Cytosine to uridine
dsRNA: Double-stranded RNA
dsRBDs: DsRNA binding domains
NLS: Nuclear localization sequence
IFN: Interferon
NES: Nuclear export sequence
MDA5: Melanoma differentiation-associated gene 5
Z-RNA: Z-type dsRNA
ZBP1: Z-DNA binding protein 1
3' UTRs: 3' Untranslated regions
miRNAs: MicroRNAs
lncRNAs: Long ncRNAs
NAs: Endogenous nucleic acids
PRRs: Pattern recognition receptors
DCs: Dendritic cells
DAMPs: Damage-associated molecular patterns
PAMPs: Pathogen-associated molecular patterns
ISG: IFN-stimulated genes
SINEs: Short interspersed nuclear elements
MDA5: Melanoma differentiation-associated gene 5
RIPK3: Receptor-interacting-serinethreonine-protein-kinase-3
MAVS: Mitochondrial antiviral signalling protein
MEF: Mouse embryonic fibroblast
PKR: Protein kinase R
BoDV: Borna disease virus
IL-6: Interleukin-6
CXCL10: Recombinant human C-X-C motif chemokine 10
TNF: Tumor necrosis factor
NF-kb: Nuclear factor Kappa B subunit
BCL2: B cell lymphoma 2
BAX: BCL2-associated X
MLKL: Mixed lineage kinase domain-like protein
AMPA: α-Amino-3-hydroxy-5-methyl-4-isoxazole-propionicacid
SDRE: Site-directed RNA editing
gRNA: Guide RNA
ASO: Antisense oligonucleotide
λN peptide: λ-Phage N protein
arRNA: ADAR-recruiting RNA
